# Cooling-Regime Effects on the Residual Mechanical Properties, Hardness and Fracture Characteristics of Fire-Exposed Hardox Abrasion-Resistant Steels

**DOI:** 10.3390/ma19204327

**Published:** 2026-10-14

**Authors:** Özer Zeybek, Casim Yazici, Gürkan Kaya, Fatih Mehmet Özkal, Yasin Onuralp Özkılıç

**Affiliations:** 1 Department of Civil Engineering, Muğla Sıtkı Koçman University, 48000 Muğla, Türkiye; 2 Department of Construction, Ağrı İbrahim Çeçen University, 04400 Ağrı, Türkiye; 3 Department of Mechanical Engineering, Erzurum Technical University, 25100 Erzurum, Türkiye; 4 Department of Civil Engineering, Atatürk University, 25240 Erzurum, Türkiye; 5 Department of Civil Engineering, Necmettin Erbakan University, 42090 Konya, Türkiye; 6 Department of Technical Sciences, Western Caspian University, Baku 1001, Azerbaijan; 7 Department of Civil Engineering, Faculty of Engineering and Natural Sciences, Dogus University, Dudullu-Ümraniye, 34775 Istanbul, Türkiye

**Keywords:** Hardox 450, Hardox 500 Tuf, abrasion-resistant steel, post-fire behavior, residual mechanical properties, Vickers hardness, XRD, SEM, empirical residual factors

## Abstract

This study examines how cooling history affects the residual properties of 4-mm-thick Hardox 450 and Hardox 500 Tuf abrasion-resistant steels after thermal exposure. The programme includes an unheated reference at 23 °C and exposures to 300, 400, 500, 600, 800 and 1000 °C, followed by air cooling (AC), water spray cooling (WSC) or blanket cooling (BC). Room-temperature tensile tests and Vickers microhardness measurements characterize the residual response; X-ray diffraction (XRD) and scanning electron microscopy (SEM) examine the reference and 800 °C conditions. Each hardness value represents the mean of five indentations. Both grades retain most of their reference ultimate tensile strength at 300 °C, whereas strength loss becomes pronounced at 500–600 °C. After 1000 °C exposure, WSC specimens retain approximately 52.5% and 79% of the reference ultimate tensile strength for Hardox 450 and Hardox 500 Tuf, respectively, with strains at maximum stress of 3.50% and 3.79%. Local post-tensile hardness remains substantially below the reference level. The results therefore show that recovery of tensile strength does not establish restoration of the original property balance. XRD and fractography support qualitative interpretation at 800 °C but do not verify the transformation mechanism at 1000 °C. Continuous piecewise linear equations describe the measured trends within the investigated conditions; their predictive accuracy and between-specimen variability remain unestablished.

## 1. Introduction

Hardox is the SSAB trade name for a family of high-strength, abrasion-resistant steels used in equipment exposed to severe wear. The present study concerns Hardox 450 and Hardox 500 Tuf, two commercial grades whose numerical designations refer to nominal Brinell hardness levels. Hardox 500 Tuf is a distinct product developed to combine the hardness associated with the 500 class with enhanced toughness; “Tuf” is part of its commercial designation, not an ASTM or EN steel-grade symbol [1,2]. These product names should therefore not be interpreted as direct equivalents of standardized structural-steel grades. The combination of hardness, strength and fabrication properties makes Hardox steels relevant to mining, agriculture, construction, transport and quarrying applications [3,4,5,6,7,8].

Hardox 500 and Hardox 500 Tuf should also be distinguished when comparing published results. Studies of conventional Hardox 500 provide useful context on grinding, machining and thermal sensitivity [3,8], but do not establish identical post-fire behavior for Hardox 500 Tuf. Hardox 450 combines abrasion resistance with formability and weldability [9,10]. SSAB specifies quenched or quenched-and-tempered delivery conditions for the investigated products and identifies EN 10029 or EN 10051, as applicable to the product form, for dimensional tolerances [1,2]. These dimensional standards do not define an equivalent alloy designation. ASTM E8/E8M and EN ISO 6892-1, cited in the experimental section, concern tensile testing rather than material-grade equivalence.

The thermal stability of the delivered condition is important because welding and subsequent heat treatment can change the local microstructure and mechanical response. Baskutis et al. [11] examined the influence of welding additives on the fusion and heat-affected zones of Hardox 450. Studies of Hardox Extreme [12], Hardox 450 [13] and Hardox 600 [14] likewise show that the properties of welded regions depend on the welding and heat-treatment history. These results establish the sensitivity of the hardened material state to thermal processing, although welding cycles differ from the unloaded furnace exposures considered here.

Published tensile and fracture studies further illustrate the need to separate strength, tensile deformation and fracture resistance. Dzioba and Pała [15] examine the temperature dependence of strength and fracture toughness in Hardox 400, while Neimitz [16] relates ductile fracture to local stress–strain conditions and microscopic observations. Białobrzeska et al. [17] report tensile strengths above 2000 MPa in Hardox Extreme and examine directional effects. Wang et al. [18] investigate thick Hardox 450 plate and show how TiN inclusions and the surrounding matrix affect fracture development. Nicolescu et al. [9] consider coatings and ballistic performance, and Mazur and Ulewicz [19] compare the strength and fatigue behavior of steels used in semi-trailers. These investigations provide useful material-specific context, but their different grades, thicknesses, loading conditions and thermal histories prevent direct transfer of their numerical results to post-fire Hardox 450 and Hardox 500 Tuf coupons.

Our recent work [20] examined the post-fire degradation and cooling response of Miilux 450 wear-resistant steel. That study considered a single Miilux 450 grade subjected to air, water-immersion, and CO_2_ fire-extinguisher cooling, whereas the present work compares two Hardox grades under air, water-spray, and insulated blanket cooling and additionally examines their XRD response. For Hardox 450 and Hardox 500 Tuf, the corresponding relationship between cooling history and the residual balance of mechanical properties remains insufficiently characterized. Existing studies largely concern wear, machining, welding, or mechanical performance outside a post-fire setting. A central question is whether rapid cooling after severe thermal exposure can recover tensile strength while leaving hardness and deformation capacity substantially altered. Comparing two Hardox grades under the same heating and cooling procedures provides a means of examining this question without attributing differences to dissimilar test protocols. The present investigation therefore combines tensile properties, microhardness, XRD, and fracture-surface observations, and develops residual-factor equations for the tested thermal histories. The objectives of this study are to:

➢ Evaluate the residual tensile properties of Hardox 450 and Hardox 500 Tuf steels after thermal exposure;
➢ Quantify the influence of AC, WSC, and BC regimes on residual strength, stiffness, strain capacity, and hardness;
➢ Interpret the observed trends using XRD patterns and SEM fracture-surface observations;
➢ Develop empirical residual-factor equations to describe the measured post-fire property trends.

The contribution of this study is a comparison of the residual strength, strain capacity and local hardness of two abrasion-resistant grades under a common nominal thermal programme. The combined measurements test whether recovery of one property is accompanied by recovery of the others. The measured trends provide a basis for further experimental validation.

## 2. Experimental Investigation

The investigated Hardox 450 and Hardox 500 Tuf steels were commercial abrasion-resistant products manufactured by SSAB. Their specified delivery conditions are quenched or quenched and tempered [1,2]. According to the EN 10204 Type 3.1 inspection certificates, the Hardox 450 plate had an average hardness of 446 HB, while the Hardox 500 Tuf plate had an average hardness of 494 HB. These certificate values are distinct from the Vickers microhardness measurements performed in the present investigation.

### 2.1. Specimen Details

**Figure 1:**
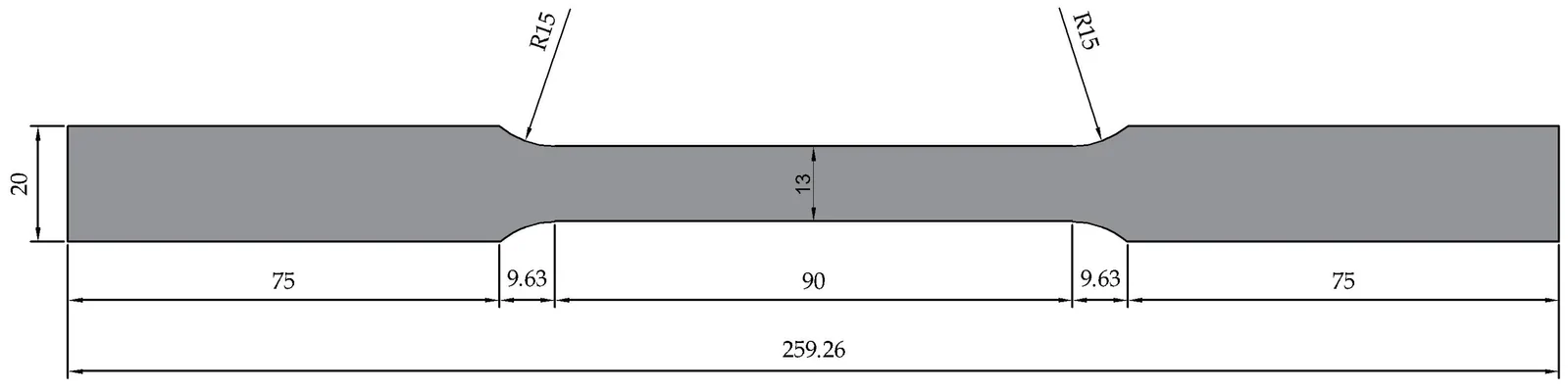
Geometric dimensions of the tensile coupon specimens (mm).

Flat dog-bone specimens were extracted from nominally 4-mm-thick Hardox 450 and Hardox 500 Tuf plates for tensile testing with reference to ASTM E8/E8M-09 [21] and EN ISO 6892-1:2019 [22]. Figure 1 gives the nominal coupon geometry, including the 90-mm parallel reduced section. This dimension should not be confused with the specimen-specific initial distance between the video-extensometer marks. Oxide scale was removed after thermal exposure and cooling, and the width and thickness of every specimen were measured using a micrometer before tensile testing. The chemical compositions in Table 1 are taken from the manufacturer’s inspection report.

### 2.2. Test Program

The experimental programme comprised thermal exposure, cooling, room-temperature tensile testing, Vickers microhardness measurements, XRD and SEM fractography.

#### 2.2.1. Heating and Cooling Treatment

Reference specimens were maintained at 23 °C. The remaining specimens were heated in a PROTHERM temperature-controlled laboratory furnace to nominal target temperatures of 300, 400, 500, 600, 800 and 1000 °C, at a programmed heating rate of 20 °C/min, followed by a 30-min holding period. A K-type thermocouple inside the furnace monitored the furnace temperature. This measurement does not provide a specimen-specific cooling history after removal from the furnace.

**Figure 2:**
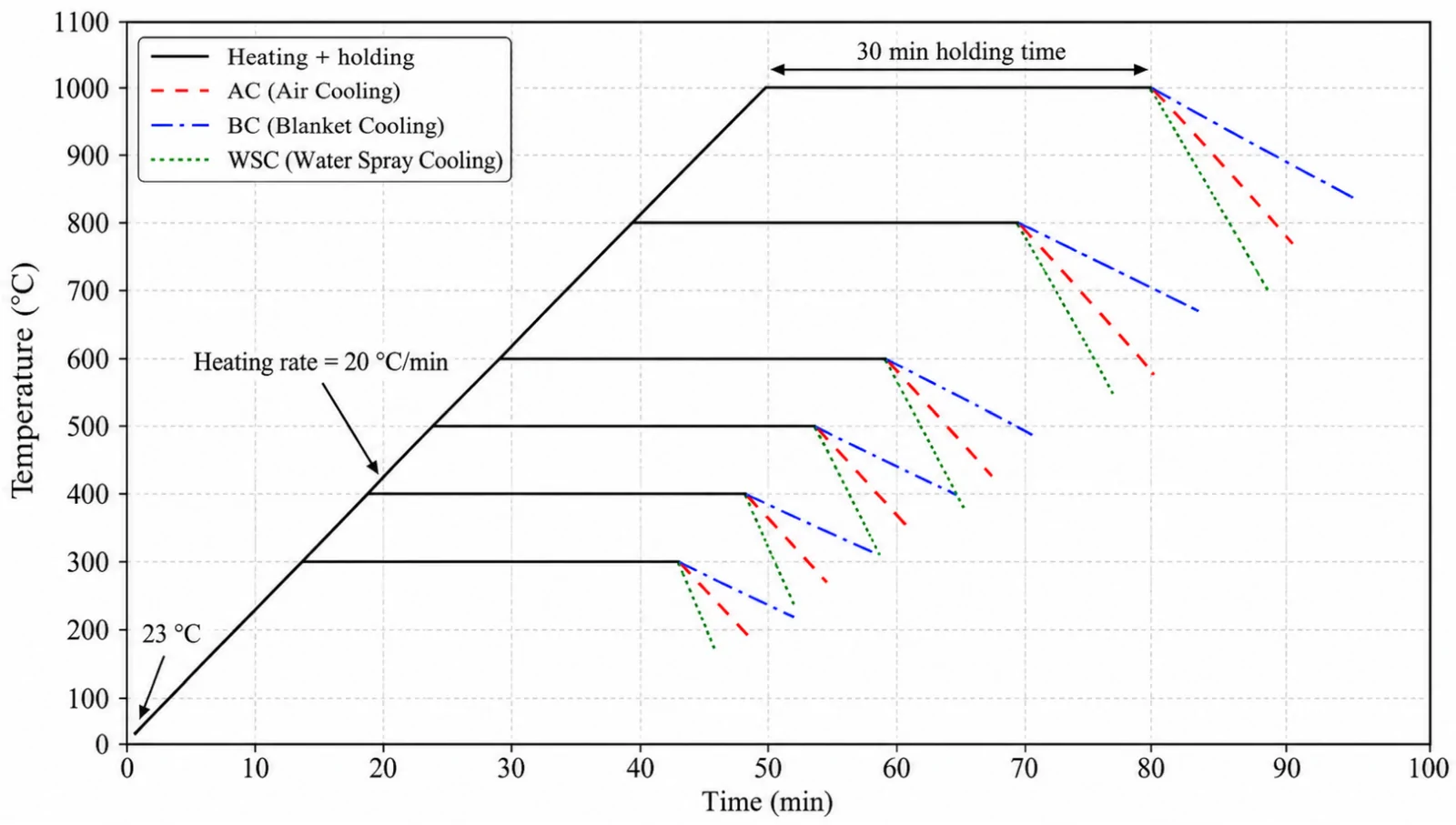
Schematic time-temperature profiles for the heating, holding, and cooling processes.

After the holding period, specimens were removed and subjected to air cooling (AC), water spray cooling (WSC) or blanket cooling (BC). For WSC, water was sprayed from approximately 50 cm until the specimens returned to room temperature; spraying lasted approximately 5 min. The pump flow rating was 80 L/min. This equipment rating is not a calibrated measurement of nozzle discharge or of the water flux reaching the specimen. No specimen-resolved cooling curves are presented; Figure 2 shows the intended sequence schematically.

**Figure 3:**
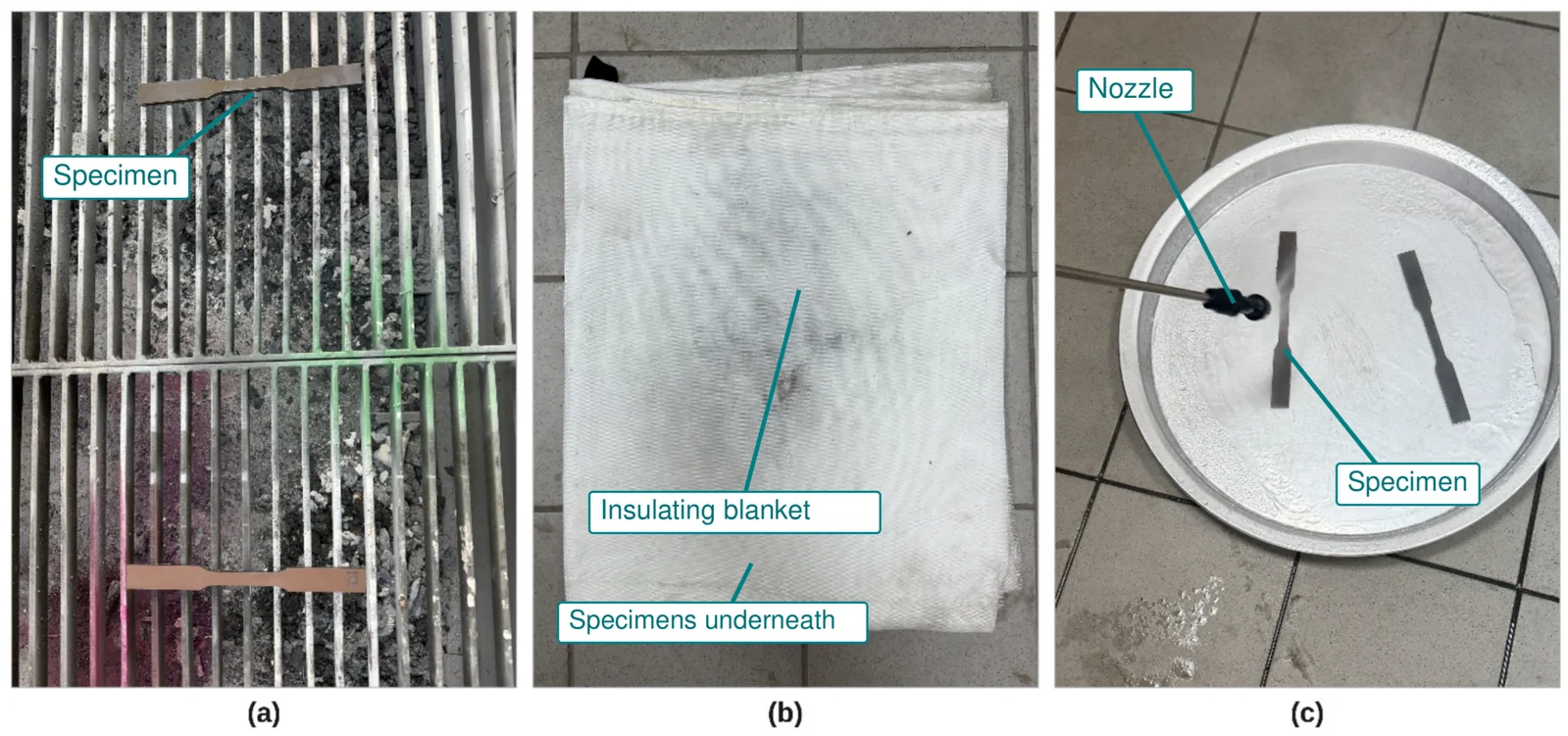
Three different cooling procedures: (**a**) AC; (**b**) BC; (**c**) WSC.

Figure 3 identifies the specimens, spray nozzle and insulating blanket. These photographs illustrate the cooling arrangements and are not calibrated dimensional views; nominal coupon dimensions are given in Figure 1. AC denotes natural cooling under ambient conditions, WSC denotes spray cooling, and BC denotes insulated cooling under a thermal blanket. The procedures impose different heat-extraction conditions, but no quantitative ranking of cooling rates is inferred from the schematic curves. Water temperature, calibrated nozzle discharge and the blanket’s thermal properties are not quantified in the present dataset, which limits exact reproduction of the cooling boundary conditions.

The cooling regimes represent idealized post-fire conditions. Their labels describe the procedures used for the 4-mm coupons; they do not define universal cooling rates for components of different thickness, geometry, or exposure conditions.

#### 2.2.2. Tensile Testing

After cooling, tensile tests were performed at room temperature using an INSTRON 5982 testing machine and a video extensometer. The testing machine undergoes annual calibration. A constant crosshead displacement rate of 1 mm/min was used. Extensometer marks were applied separately to each specimen, and the corresponding initial marked length was recorded in its raw test file. Engineering strain was obtained from the change in that marked length. If L_0,i_ is the initial marked length of specimen *i* in millimeters, the crosshead-based nominal strain-rate estimate is 1/(60*L*_0,*i*_) s^−1^. This estimate is specimen-dependent and should not be equated with a constant measured gauge strain rate in a displacement-controlled test.

The stress–strain curves were used to determine elastic modulus (E), 0.2% proof stress (f_y_), ultimate tensile strength (f_u_) and strain at maximum engineering stress (ε_u_). The reported strain parameter includes the strain present at maximum load and is not elongation at fracture.

#### 2.2.3. Hardness and Microstructural Investigation

**Figure 4:**
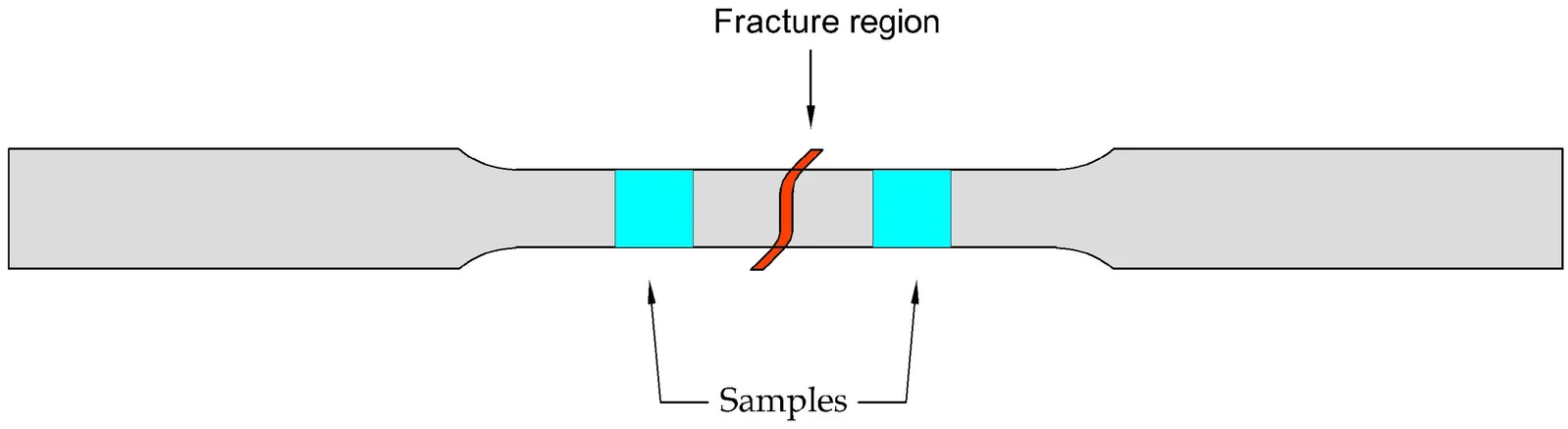
Characterization of the sample collection areas.

After tensile testing, small specimens were extracted from the regions adjacent to the fracture zone shown in Figure 4. These regions were used for microhardness measurements and, for the selected conditions, XRD. SEM examined the tensile-fracture surfaces. The 800 °C condition was selected because the tensile response showed pronounced changes in strength and strain capacity at this temperature; both grades and all three cooling regimes were examined alongside the unheated references. No XRD, SEM or optical-metallographic characterization was performed at 1000 °C.

Microhardness was measured using a SHIMADZU HMV-G20 tester after surface preparation by grinding with abrasive paper. Five Vickers indentations were made at different locations on each sample under a 50-gf load (HV0.05) with a 10-s dwell time. The arithmetic mean of these five readings is reported in Section 3.3. Because sampling followed tensile failure and occurred near the fracture zone, the results may reflect local plastic strain and surface condition in addition to thermal exposure; they should not be treated as an undeformed bulk-hardness measurement.

XRD measurements were performed using a GNR-Explorer diffractometer with Cu–Kα radiation (λ = 1.5418 Å), in a symmetric scanning configuration over 2θ = 10–90°. The scan rate was 0.05°/s; this value denotes angular scanning speed, not the angular step interval. The patterns were examined using Match software. The analysis is qualitative: no Rietveld phase quantification, EBSD analysis or quantitative metallography is reported, and repeat-scan precision is not evaluated.

Fracture surfaces were examined using a QUANTA-FEG 250 scanning electron microscope. The instrument scale bars are retained, and their values and acquisition magnifications are repeated in the figure captions. Morphological labels identify observable features; they do not establish inclusion chemistry, phase fractions or metallurgical grain size.

### 2.3. Replication and Measurement Uncertainty

One independent tensile specimen was tested per steel–temperature–cooling condition, together with one unheated reference per grade: 18 heated specimens and one reference for each grade, giving 38 tensile tests in total. Consequently, between-specimen standard deviations, standard errors, confidence intervals and significance tests cannot be obtained for the tensile results. The hardness error bars defined in Section 2.2.3 describe dispersion among indentation locations within a specimen, rather than independent thermal-treatment replicates. XRD and SEM provide qualitative characterization of the selected specimens.

Figure 1 reports nominal geometry, whereas individual coupon widths and thicknesses were measured after scale removal. Annual machine calibration and specimen-specific dimensional measurements do not, by themselves, define the uncertainty of a derived mechanical property. A complete propagated uncertainty budget for force, strain, dimensions and temperature is not reported. Nominal process settings and certificate composition values are therefore not supplemented with assumed ± ranges. Small differences between conditions require particular caution, especially for elastic modulus and local hardness.

## 3. Results and Discussion

### 3.1. Stress–Strain Response

**Figure 5:**
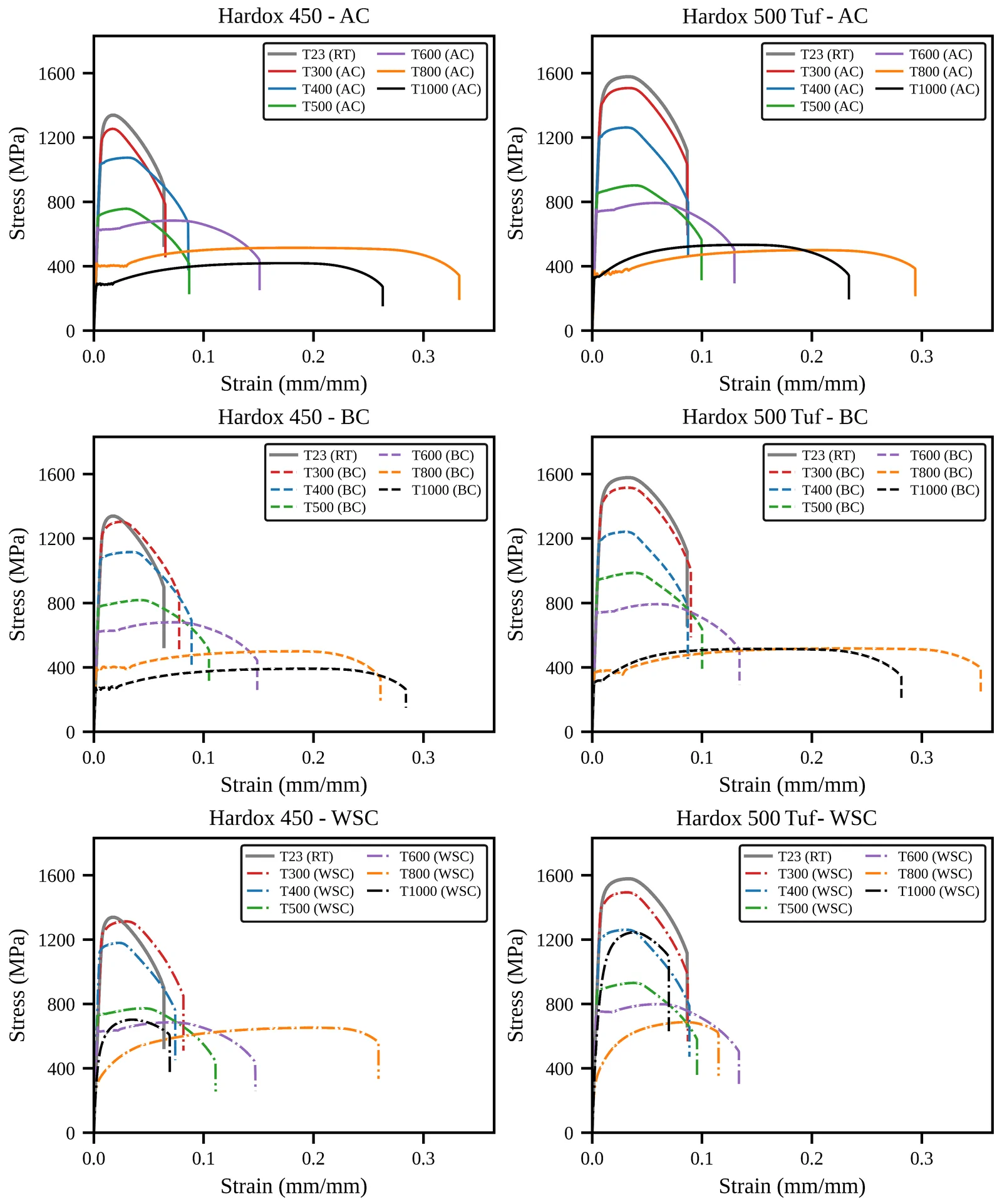
Stress–strain curves of Hardox 450 and Hardox 500 Tuf steel.

The engineering stress–strain curves for Hardox 450 and Hardox 500 Tuf tested at room temperature after the specified thermal exposure and cooling procedure are presented in Figure 5. The comparisons below describe the observed responses; differences between conditions are not interpreted as statistically established treatment effects.

In the as-received condition, Hardox 500 Tuf exhibited a higher ultimate tensile strength than Hardox 450, approximately 1579 and 1340 MPa, respectively. Both grades reached maximum engineering stress at relatively small strains. Heating generally reduced proof stress and tensile strength, while the strain at maximum stress increased in many conditions, particularly after AC and BC at 800–1000 °C. This comparison concerns the strain attained at peak load and does not imply that total elongation or fracture toughness follows the same trend.

A distinct yield plateau was not observed in the as-received specimens or in several thermally exposed specimens. Therefore, the 0.2% proof stress (*f_y_*) was adopted as the yield strength parameter. The residual properties extracted from the stress–strain curves were the elastic modulus (E), 0.2% proof stress (*f_y_*), ultimate tensile strength (*f_u_*), and strain at ultimate tensile strength (*ε_u_*). Here, *ε_u_* describes the strain attained at the maximum engineering stress and should be distinguished from elongation at fracture.

### 3.2. Residual Strength, Strain and Stiffness

The residual factors permit comparison of the fractional changes in the two grades, despite their different reference strengths. They should also be considered alongside the absolute stress values, because equal retention factors do not imply equal residual load-bearing capacity.

**Figure 6:**
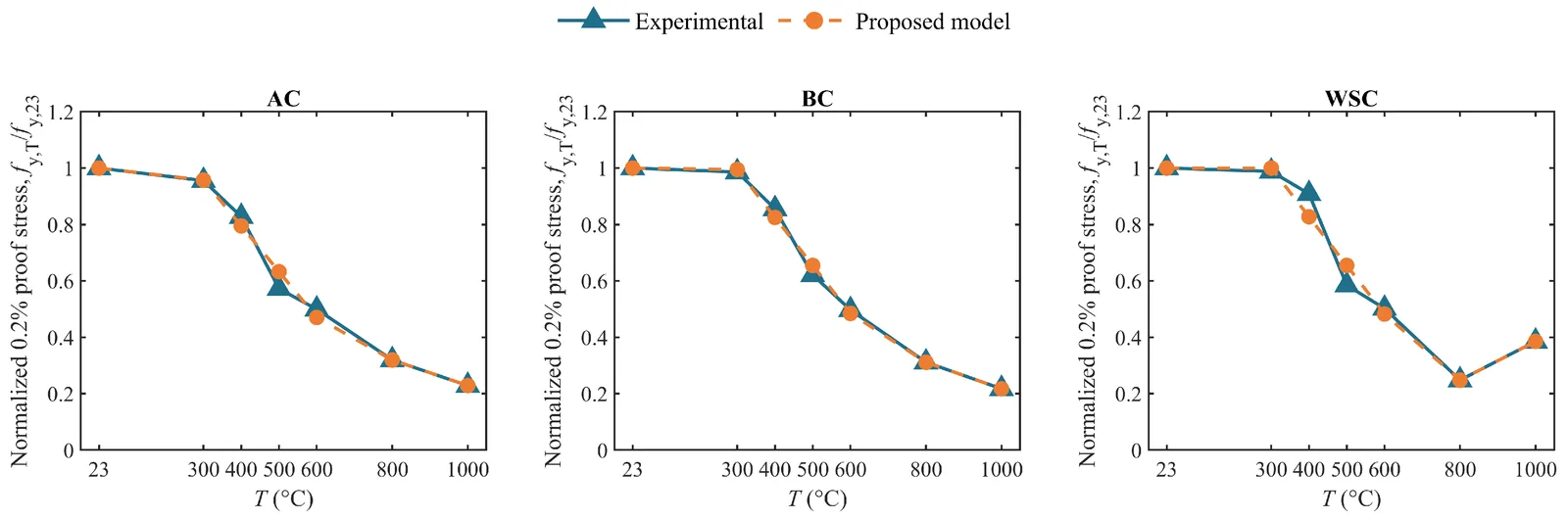
Normalized 0.2% proof stress of Hardox 450 under different cooling methods.

**Figure 7:**
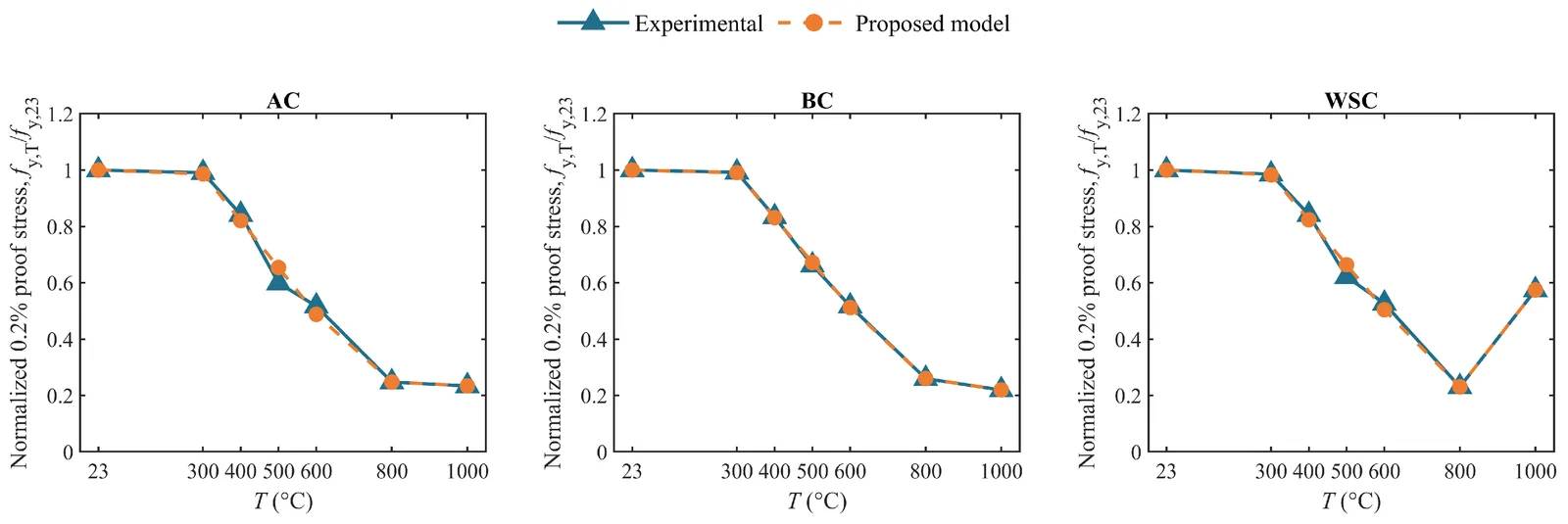
Normalized 0.2% proof stress of Hardox 500 Tuf under different cooling methods.

**Figure 8:**
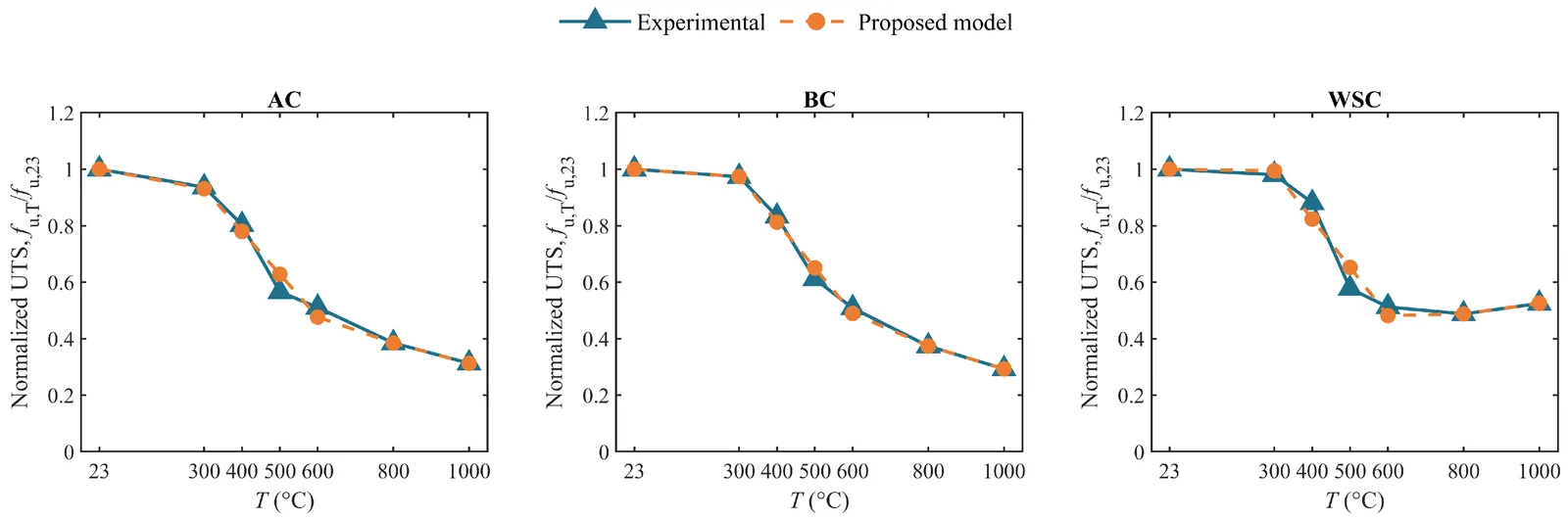
Normalized ultimate tensile strength (UTS) of Hardox 450 under different cooling methods.

**Figure 9:**
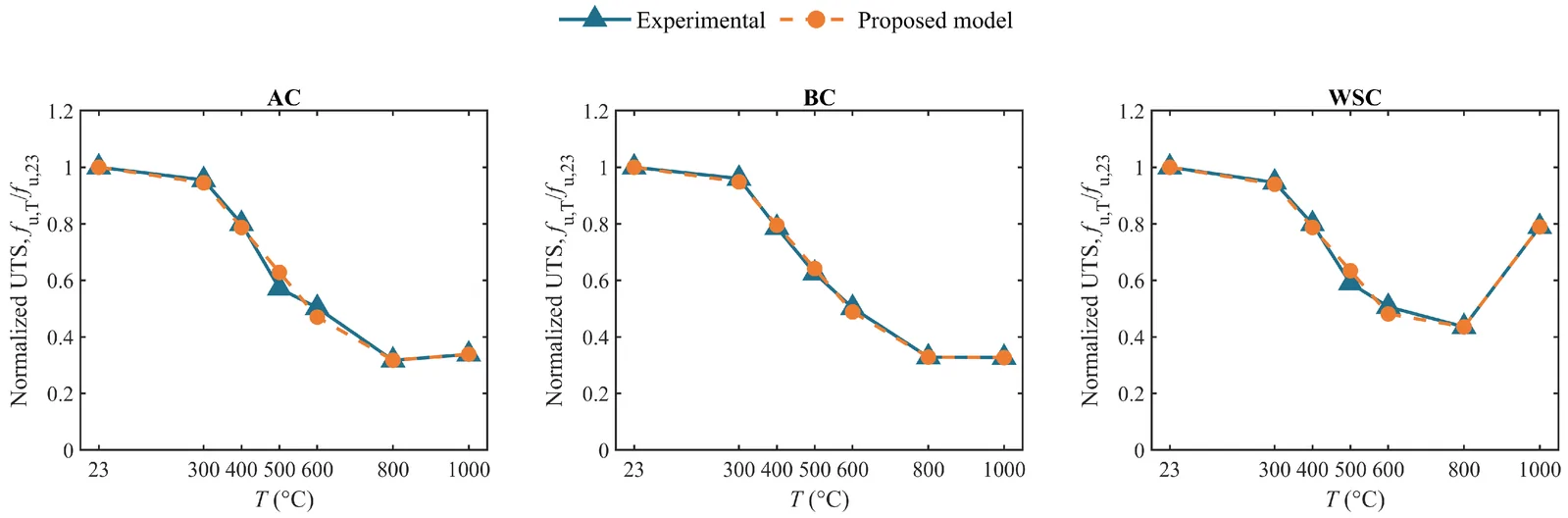
Normalized ultimate tensile strength (UTS) of Hardox 500 Tuf under different cooling methods.

**Figure 10:**
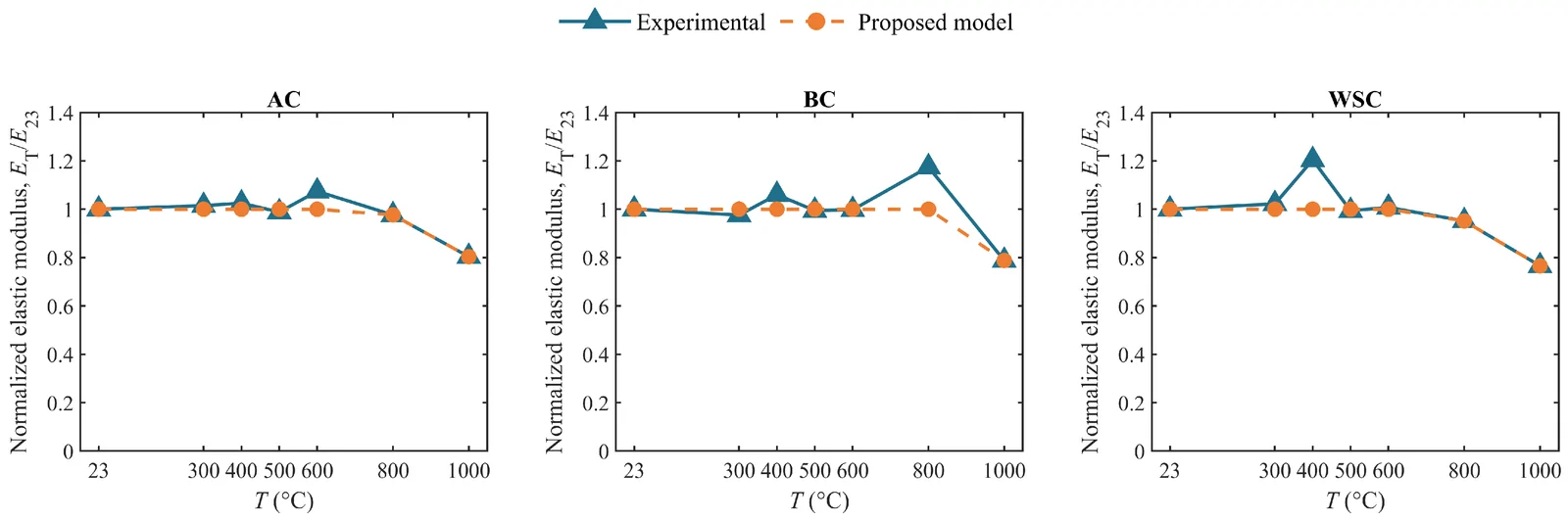
Normalized elastic modulus of Hardox 450 under different cooling methods.

**Figure 11:**
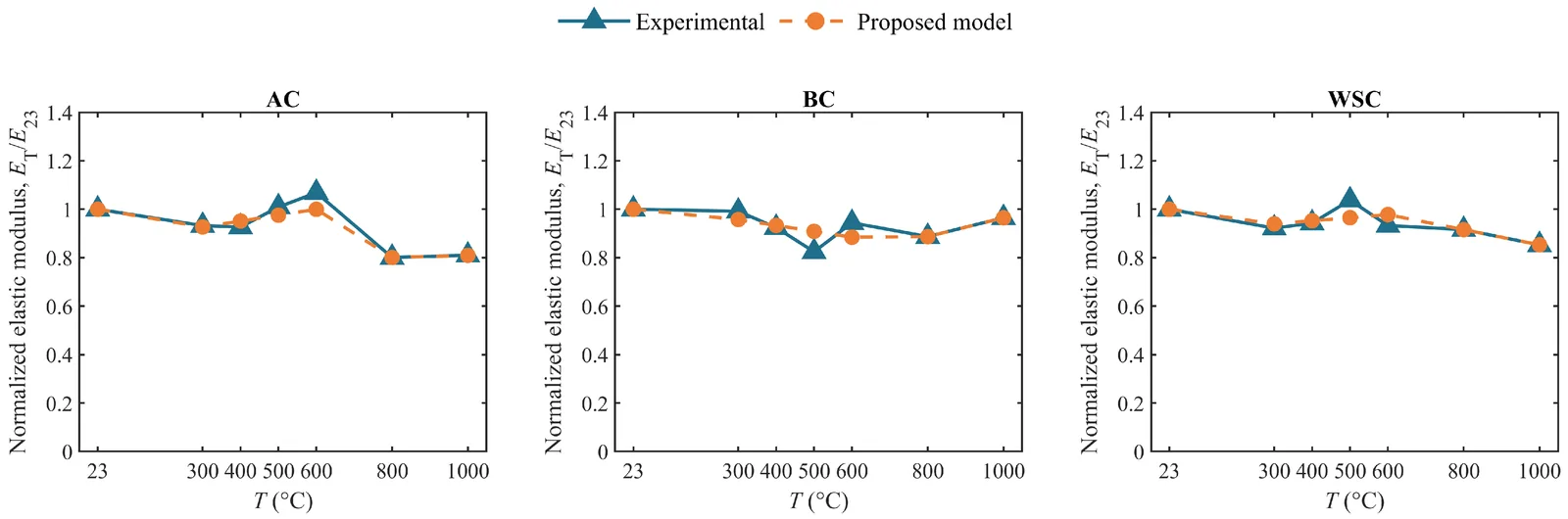
Normalized elastic modulus of Hardox 500 Tuf under different cooling methods.

**Figure 12:**
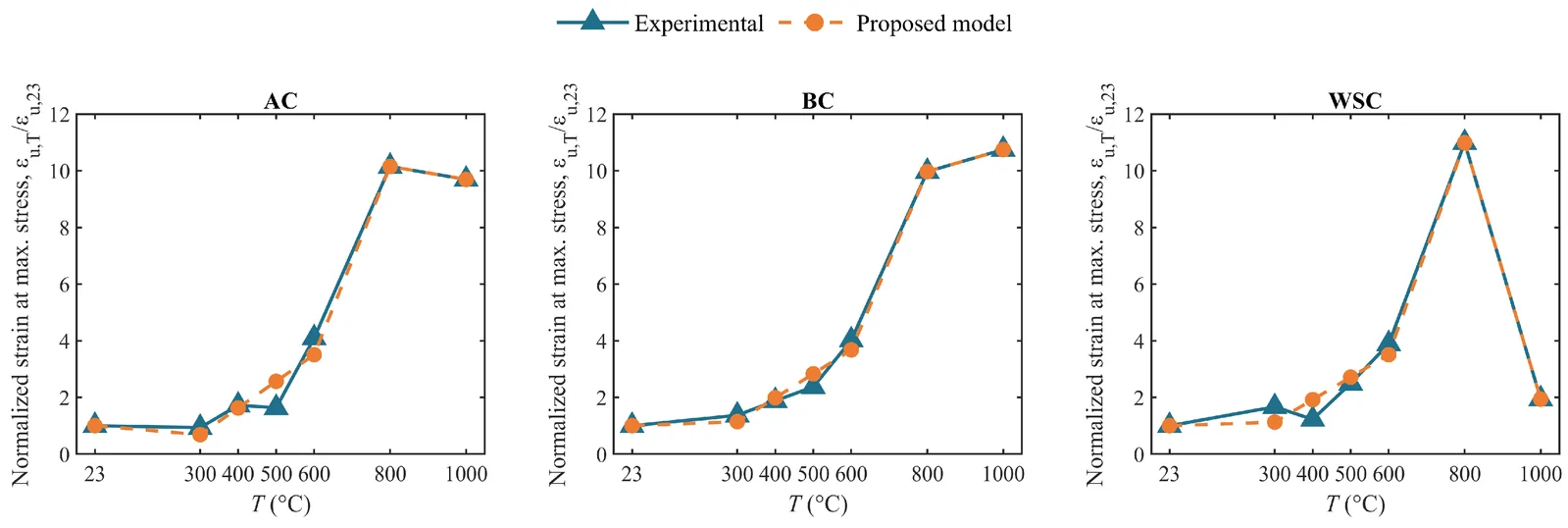
Normalized strain at maximum engineering stress of Hardox 450 under different cooling methods.

**Figure 13:**
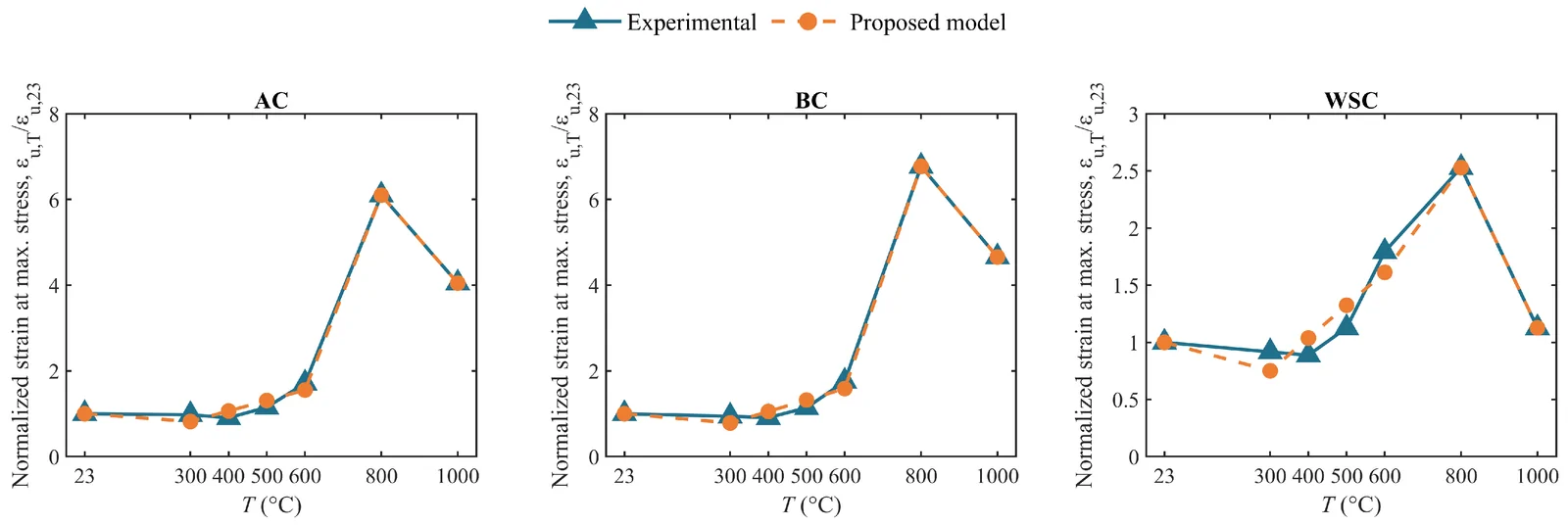
Normalized strain at maximum engineering stress of Hardox 500 Tuf under different cooling methods.

The residual factors in Figure 6, Figure 7, Figure 8, Figure 9, Figure 10, Figure 11, Figure 12 and Figure 13 are *E_T_*/*E*_23_, *f_y_*_,T_/*f_y_*_,23_, *f_u_*_,T_/*f_u_*_,23_ and *ε_u_*_,T_/*ε_u_*_,23_. The subscript *T* identifies the peak exposure temperature before cooling, while _23_ denotes the unheated reference. All tensile properties are measured at room temperature. Figure 6, Figure 7, Figure 8 and Figure 9 show strength factors, Figure 10 and Figure 11 show modulus factors, and Figure 12 and Figure 13 show strain-at-maximum-stress factors. The common reference denominator also carries unquantified experimental uncertainty.

The normalized residual factors clearly demonstrate the combined influence of peak exposure temperature and cooling method on the post-fire mechanical response of Hardox 450 and Hardox 500 Tuf. For both steels, exposure to 300 °C caused only limited degradation in ultimate tensile strength. Hardox 450 retained approximately 94–98% of its room-temperature UTS, while Hardox 500 Tuf retained approximately 95–96%. At 400 °C, a more pronounced reduction was observed, with UTS retention decreasing to approximately 80–88% for Hardox 450 and to 79–80% for Hardox 500 Tuf. This indicates the onset of noticeable thermal softening.

This retention of UTS at 300 °C should not be interpreted as a permissible service-temperature limit. The manufacturer cautions that the complete delivered property set is not retained after elevated-temperature exposure [1,2]. Retention of tensile strength alone does not establish the preservation of impact behavior, wear resistance or the original hardened condition.

Strength loss became more pronounced at 500–600 °C. At 500 °C, UTS retention ranged from approximately 57% to 61% for Hardox 450 and from 57% to 63% for Hardox 500 Tuf. At 600 °C, both grades retained approximately 50–51% of the reference UTS. The small separation between cooling conditions at this temperature is consistent with substantial softening during heating and holding. This proximity does not, by itself, establish statistical equivalence between cooling methods.

The limited cooling sensitivity at 500–600 °C is consistent with softening acquired during heating and holding. Recovery of the dislocation structure and changes in the carbide population are plausible contributions in the initially hardened matrix. Related thermal sensitivity has been reported in Hardox heat-treatment and welding studies [23,24]. Once these strengthening features have changed, cooling to room temperature need not restore the original strength. The present results support this interpretation of the mechanical response, although dislocation density and carbide size were not measured and their separate contributions cannot be quantified.

At 800 and 1000 °C, the influence of the cooling regime became much more pronounced. Under AC and BC conditions, both Hardox 450 and Hardox 500 Tuf exhibited severe degradation of residual strength and a considerable increase in strain capacity, indicating pronounced thermal softening. In contrast, WSC promoted partial recovery of tensile strength, with the effect being most evident after exposure to 1000 °C. For Hardox 450, the UTS after 1000 °C exposure increased from 419.5 MPa under AC and 392.3 MPa under BC to 703.2 MPa under WSC, which corresponds to approximately 52.5% of the room-temperature value. This recovery was much more pronounced for Hardox 500 Tuf, where the UTS increased from 533.8 MPa under AC and 515.8 MPa under BC to 1246.2 MPa under WSC, corresponding to approximately 79% of the room-temperature value. A qualitatively similar cooling-dependent response was reported for Miilux 450 [20], for which water cooling above 700 °C produced substantially greater strength recovery than slower cooling methods, but at the expense of ductility. However, the water-immersion cooling used for Miilux 450 differs from the water-spray procedure adopted here; therefore, the agreement should be interpreted in terms of the general effect of rapid cooling rather than as a direct quantitative correspondence.

At the higher exposure temperatures, transformation during heating and subsequent cooling becomes a plausible additional source of the differences between AC, BC, and WSC. Faster heat extraction can alter the transformation products formed from austenite and thereby increase residual strength. Gallina et al. [23] found that controlled quenching and tempering could increase Hardox 500 hardness, whereas normalization and annealing produced softer states. The present WSC procedure was not an equivalent controlled heat treatment: the recovery was incomplete and differed between tensile strength and hardness. It should therefore be interpreted as a response to the imposed thermal history, without assuming restoration of the manufacturer’s microstructure.

The stronger WSC response of Hardox 500 Tuf also warrants comparison with the compositions in Table 1. This grade contains more carbon, chromium, and nickel than the investigated Hardox 450 plate, but less manganese. These differences can influence transformation behavior and the strength of the resulting matrix, so the two grades need not respond equally to the same nominal cooling procedure. However, composition alone cannot identify the mechanism. The initial processing condition and the actual specimen cooling histories also matter, and no quantitative phase balance is available to separate these effects.

The higher WSC strength at 1000 °C was accompanied by a lower strain at maximum stress than in the AC and BC specimens. For Hardox 500 Tuf, εu was 13.60% under AC, 15.65% under BC and 3.79% under WSC; the corresponding Hardox 450 values were 17.47%, 19.36% and 3.50%. These individual results indicate a strength–strain-capacity trade-off under the tested conditions. They do not establish a quantitative relationship between cooling rate and ductility, because cooling rates and between-specimen scatter were not determined.

For component assessment, this reduction in strain at UTS indicates less deformation before the maximum tensile load is reached. It does not establish a corresponding reduction in fracture toughness or impact energy, which were not measured. The distinction matters because a steel can display dimpled fracture locally while still reaching its peak engineering stress at a relatively small overall strain. Residual strength and resistance to deformation localization therefore answer different questions about post-fire performance.

The measured elastic-modulus factors varied less than the strength factors and did not follow a consistently monotonic temperature dependence. For Hardox 450, they ranged approximately from 0.77 to 1.20; for thermally exposed Hardox 500 Tuf, they ranged approximately from 0.80 to 1.07. Up to 600 °C, the apparent modulus was 202 ± 12 GPa for Hardox 450 and 194 ± 13 GPa for Hardox 500 Tuf (mean ± standard deviation over the reference and the twelve heated specimens of each grade), with no resolvable dependence on exposure temperature or cooling method. Individual values as high as 237 GPa (Hardox 450, WSC, 400 °C) and 231 GPa (Hardox 450, BC, 800 °C) are not physically plausible for steel and indicate the limits of the strain measurement at low stress. After 1000 °C, five of the six specimens gave 151–173 GPa; whether this reflects the material or specimen distortion after cooling cannot be established from the present tests. Local increases above unity and differences between adjacent temperatures cannot be separated from experimental scatter.

The different responses of modulus and strength show that one residual factor cannot represent all aspects of the constitutive behavior. Retention of an approximately unchanged elastic slope does not imply retention of the stress required for plastic deformation. Conversely, the small increases above the reference modulus in some conditions should not be interpreted as evidence of beneficial thermal treatment without an uncertainty assessment. Elastic-slope estimates depend on the strain-measurement accuracy and the fitting interval, making repeatability particularly relevant when the observed changes are modest.

The results distinguish a temperature range dominated by strength loss from a higher-temperature range in which cooling history strongly alters the balance of properties. This distinction is relevant to the assessment of fire-exposed components: a relatively high residual UTS after rapid cooling does not establish that the component has retained its original deformation capacity. Conversely, the larger strain capacity after AC or BC accompanies substantial losses in strength. A reuse assessment therefore requires the residual proof stress and strain capacity to be considered together with the demands on the component.

### 3.3. Hardness Results

**Figure 14:**
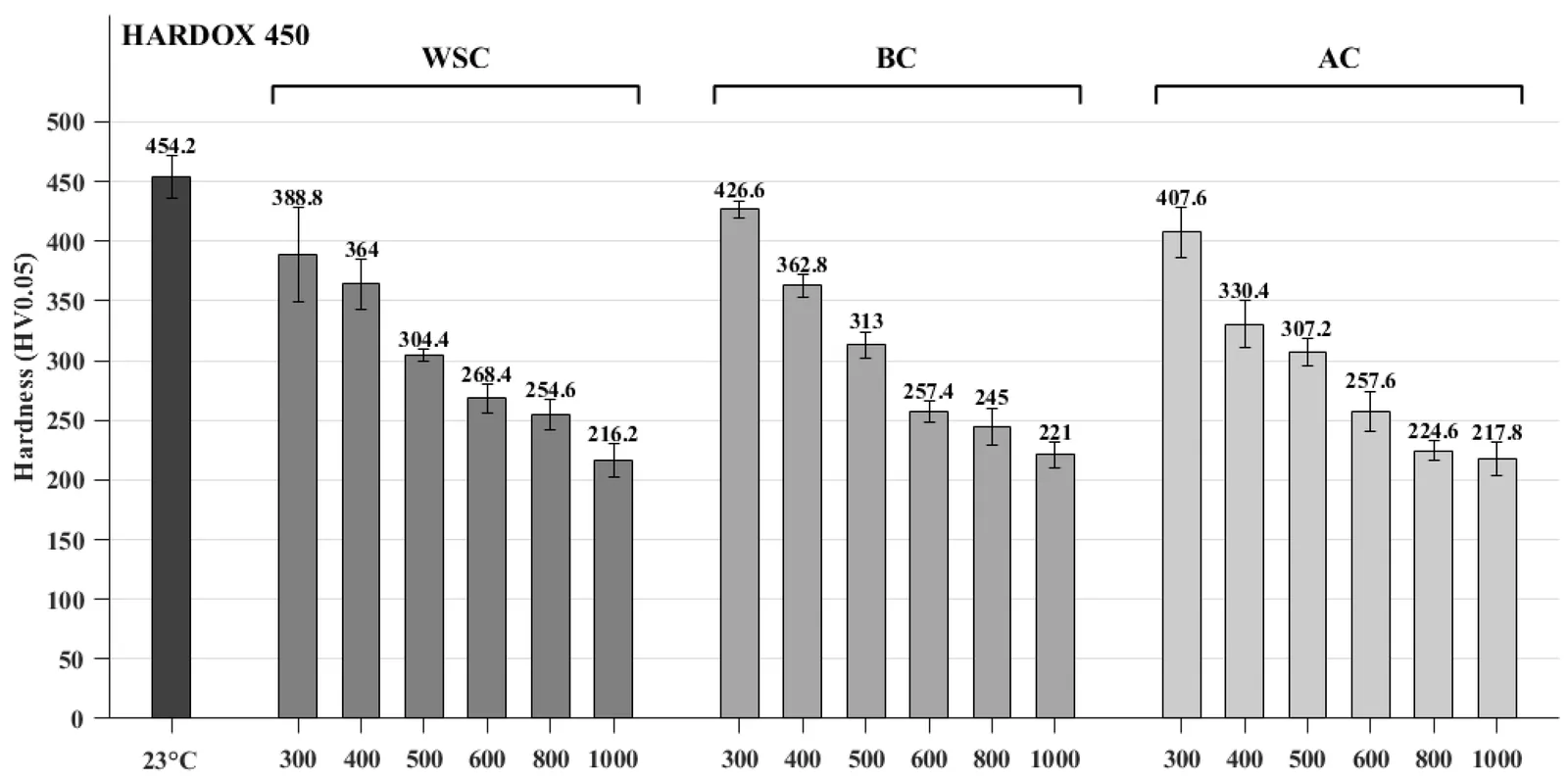
Hardness results of Hardox 450 specimens under different thermal exposure and cooling conditions.

**Figure 15:**
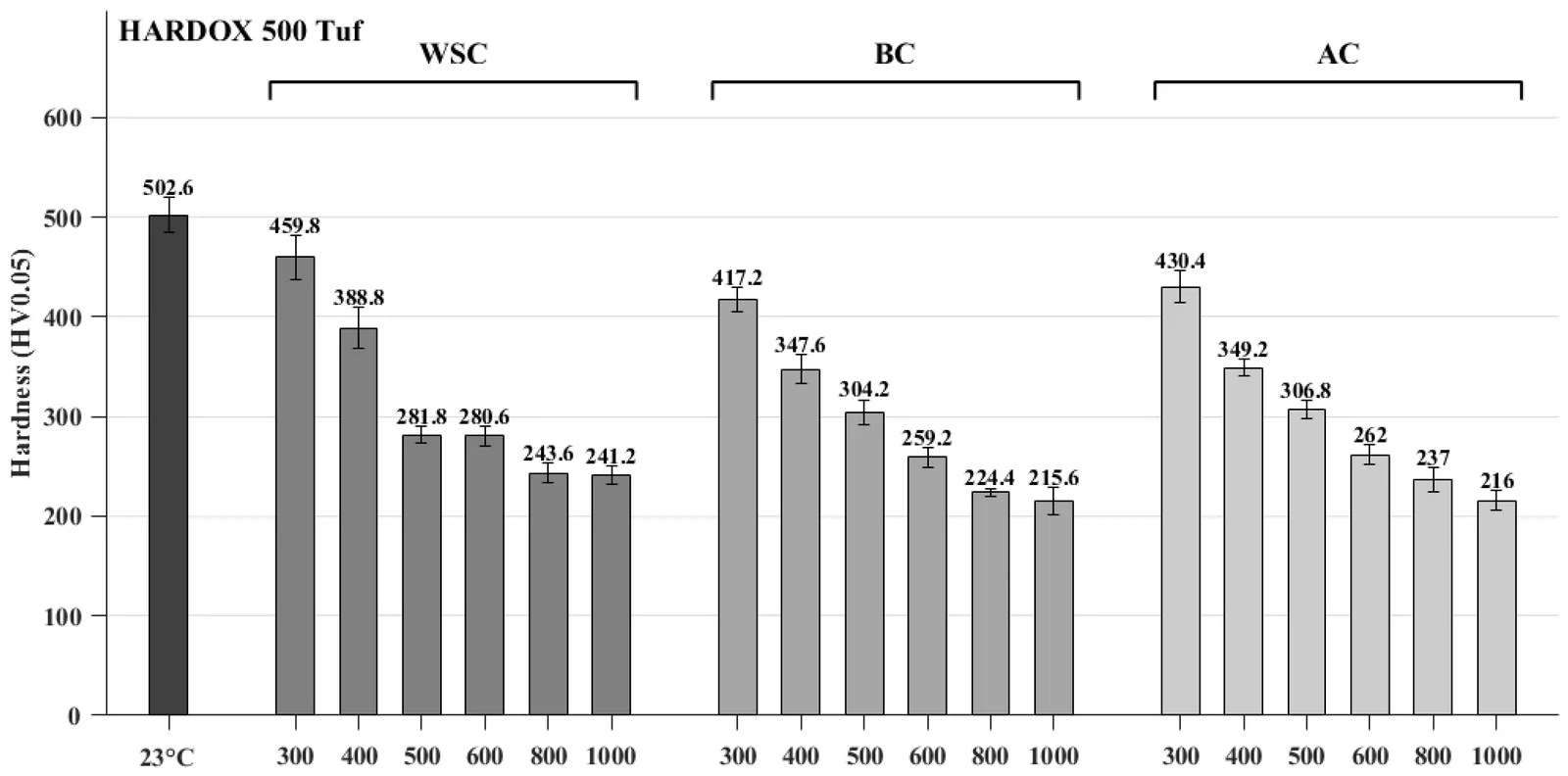
Hardness results of Hardox 500 Tuf specimens under different thermal exposure and cooling conditions.

Hardox steels derive their high hardness and strength from their composition and hardened microstructure [23,25,26]. Thermal exposure can modify this initial state, as also observed in studies of heat-treated and welded Hardox steels [23,24,27]. The hardness results in Figure 14 and Figure 15 are therefore considered alongside the tensile response to determine whether the two measurements indicate comparable levels of residual performance.

The hardness test results for Hardox 450 and Hardox 500 Tuf under different thermal exposure conditions are presented in Figure 14 and Figure 15, respectively. It should be explicitly noted that the microhardness measurements in this study were conducted near the tensile fracture region of the specimens. Consequently, the measured hardness values represent a combined influence of the microstructural transformations resulting from the thermal exposure and the localized strain hardening (plastic deformation) developed during necking and fracture under tensile loading. While these measurements effectively capture the local mechanical state of the material adjacent to failure, the potential contribution of plastic strain to the elevated hardness levels should be taken into account when evaluating the bulk hardness of the heat-treated plates.

**Figure 16:**
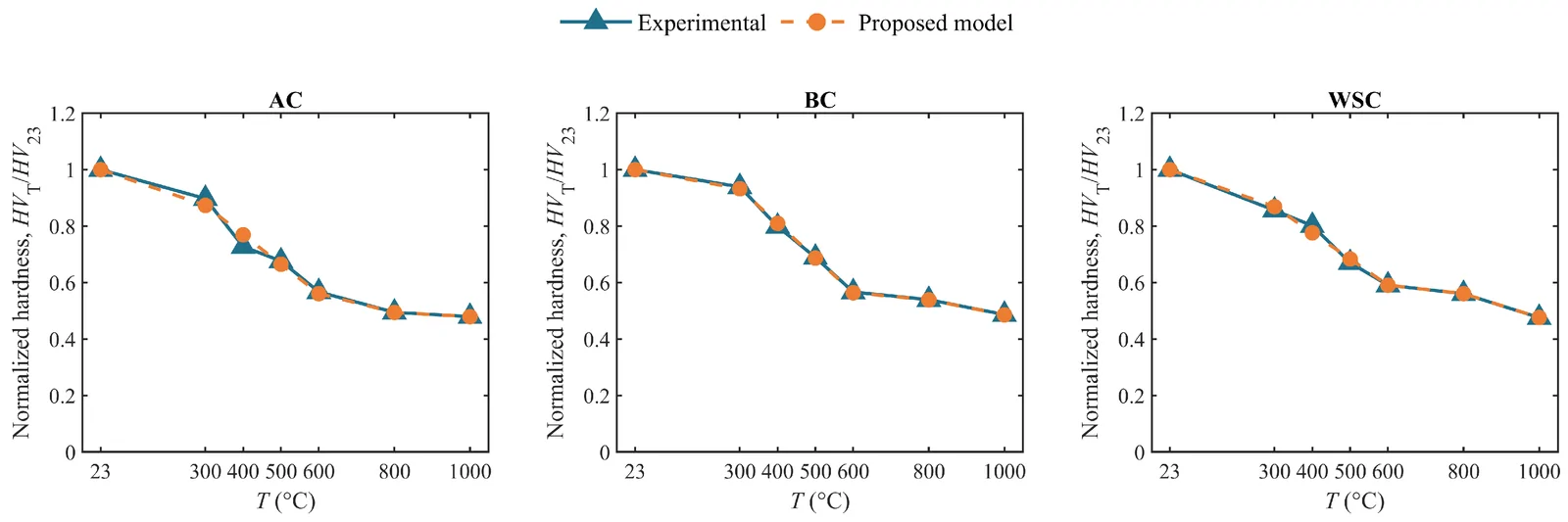
Normalized hardness of Hardox 450 under different cooling conditions.

**Figure 17:**
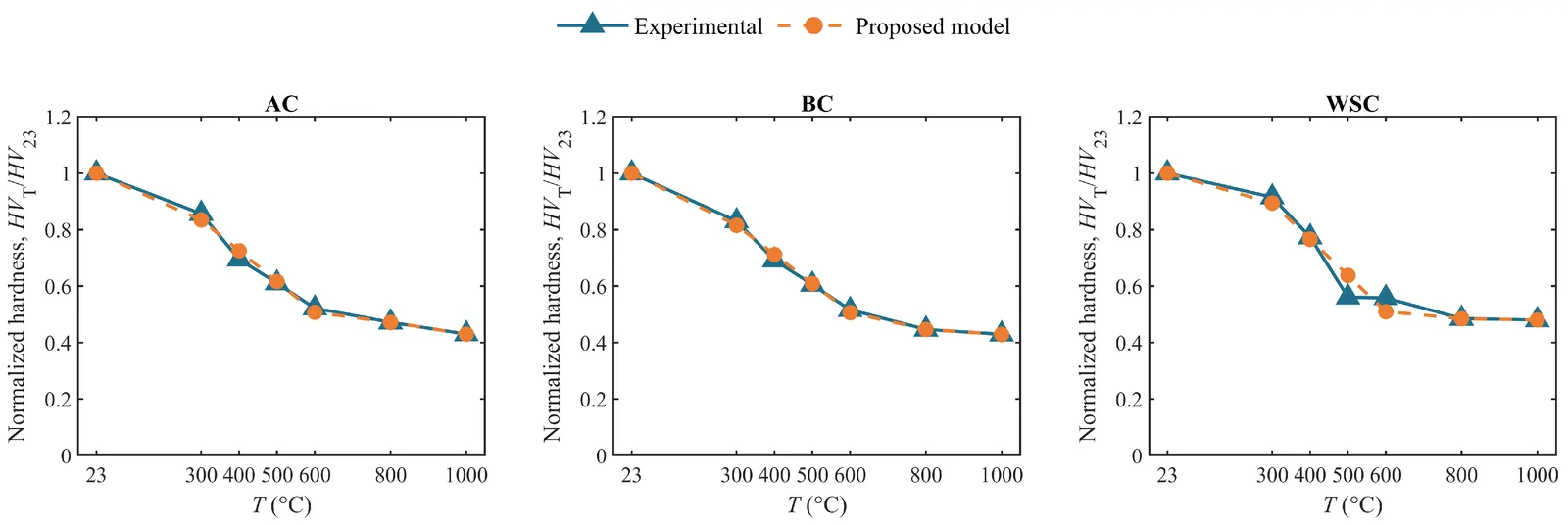
Normalized hardness of Hardox 500 Tuf under different cooling conditions.

The reference microhardness values were 454.2 HV0.05 for Hardox 450 and 502.6 HV0.05 for Hardox 500 Tuf. Figure 14 and Figure 15 show the mean hardness values, and Figure 16 and Figure 17 show the corresponding normalized values. The overall reduction with thermal exposure indicates substantial local softening of the initially hardened microstructures.

For Hardox 450, the hardness after exposure to 1000 °C was 217.8 HV under AC, 216.2 HV under WSC, and 221 HV under BC, compared with 454.2 HV in the as-received condition. The corresponding normalized values are approximately 0.480, 0.476, and 0.487. Thus, the measurements indicate retention of only about half of the reference hardness, with little separation between cooling conditions at this temperature. This contrasts with the higher residual UTS of the WSC specimen. The convergence in local hardness does not, by itself, establish identical microstructures or demonstrate that thermal softening is irreversible under every possible cooling treatment.

For Hardox 500 Tuf, the hardness decreased from 502.6 HV to 216.0 HV under AC and 215.6 HV under BC after exposure to 1000 °C. WSC gave a higher value of 241.2 HV, corresponding to approximately 48% of the reference hardness, although it remained slightly below the WSC value at 800 °C. The UTS retention of approximately 79% under the same nominal 1000 °C WSC condition therefore has no comparable recovery in the hardness measurements. This difference limits any interpretation of strength recovery as restoration of the as-received material state.

The result also differs from the post-fire Miilux 450 study [20], in which rapid water cooling produced recovery in both strength and hardness. Differences in grade, initial processing, cooling method and measurement location prevent a direct numerical comparison. The present hardness–strength divergence is therefore a feature requiring further verification, rather than evidence that rapid cooling restores the original material condition.

The hardness–strength discrepancy must be interpreted in light of both the thermal history and the sampling procedure. UTS represents the tensile gauge-section response, whereas HV0.05 probes small surface regions near the fracture zone after plastic deformation. Oxide scale was removed and specimen dimensions were measured before tensile testing, but scale removal does not demonstrate removal of any underlying decarburized layer. Decarburization depth was not measured. A relatively soft surface, local heterogeneity and prior plastic strain are therefore possible contributions to the discrepancy, not verified explanations. Neither a bulk phase fraction nor an undeformed bulk hardness can be inferred from these measurements.

For wear-resistant components, the persistent loss of hardness is relevant even where WSC increases tensile strength. The present measurements do not include wear tests, so neither an abrasion-loss rate nor a remaining service life can be deduced. They do show that tensile-strength retention cannot serve as a substitute for evaluating the surface-related properties required in service. This is particularly evident for Hardox 500 Tuf after 1000 °C WSC, where the strength and hardness residual factors differ substantially.

### 3.4. XRD Assessment

**Figure 18:**
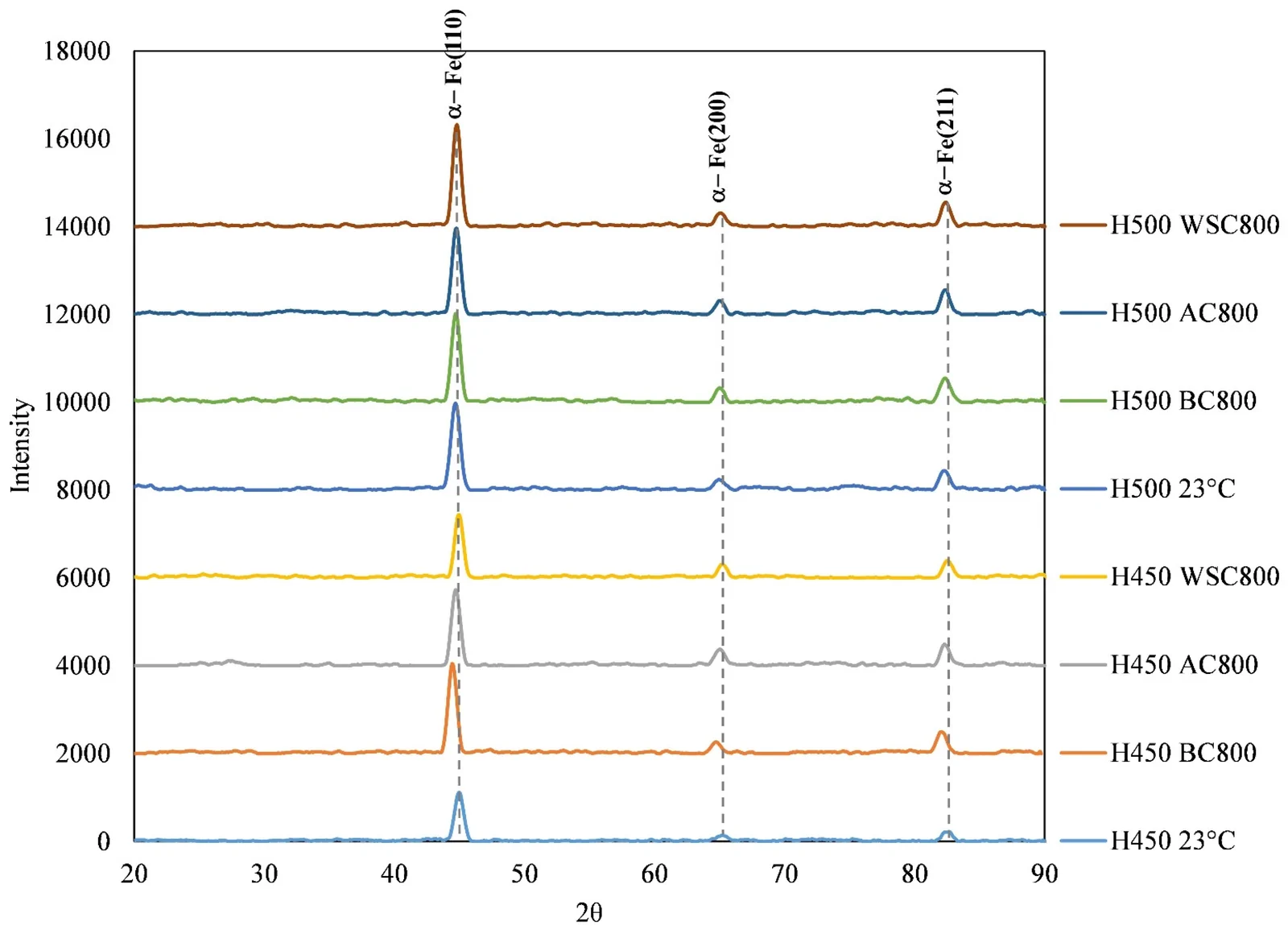
XRD patterns of Hardox 450 and Hardox 500 Tuf specimens.

The XRD patterns for the as-received specimens and those exposed to 800 °C are presented in Figure 18. The principal reflections near 2θ = 44.5°, 65°, and 82° are consistent with the (110), (200), and (211) reflections of an α-Fe-type matrix. The martensitic structures reported for these grades [27,28] provide context for interpreting the as-received material. However, these peak positions alone do not uniquely distinguish low-carbon martensite from ferritic transformation products in the heat-exposed specimens. No distinct retained-austenite peaks were resolved; this establishes no more than the absence of a detectable signal under the measurement conditions, rather than a quantitative upper bound on the austenite fraction.

The small apparent peak shifts between the as-received and 800 °C patterns are compatible with changes in lattice spacing or residual stress, arising from thermal exposure and/or prior tensile deformation; however, their origin cannot be assigned uniquely without quantitative peak fitting and a calibrated stress or lattice-parameter analysis. The patterns support comparison of the dominant diffraction features across conditions, while phase fractions and the extent of martensite formation remain unresolved. Since the presented XRD data concern 800 °C, they also cannot directly verify the transformation mechanism responsible for the strength response at 1000 °C.

### 3.5. SEM Fractography

**Figure 19:**
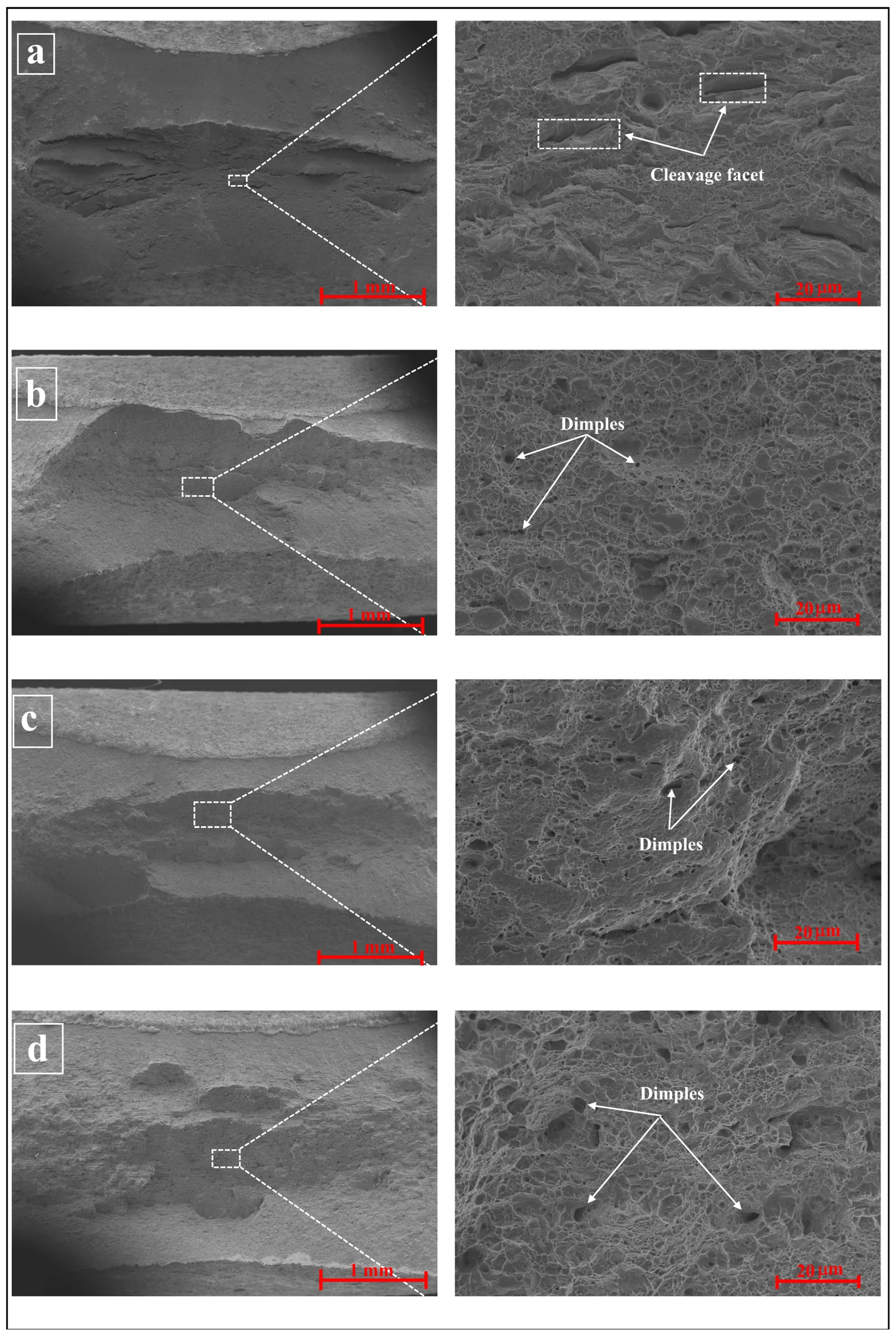
SEM images of the fracture surfaces of Hardox 450 specimens: (**a**) 23 °C, (**b**) AC 800 °C, (**c**) BC 800 °C and (**d**) WSC 800 °C. Left panels: 100×, 1-mm scale bars; right panels: 4000×, 20-µm scale bars.

**Figure 20:**
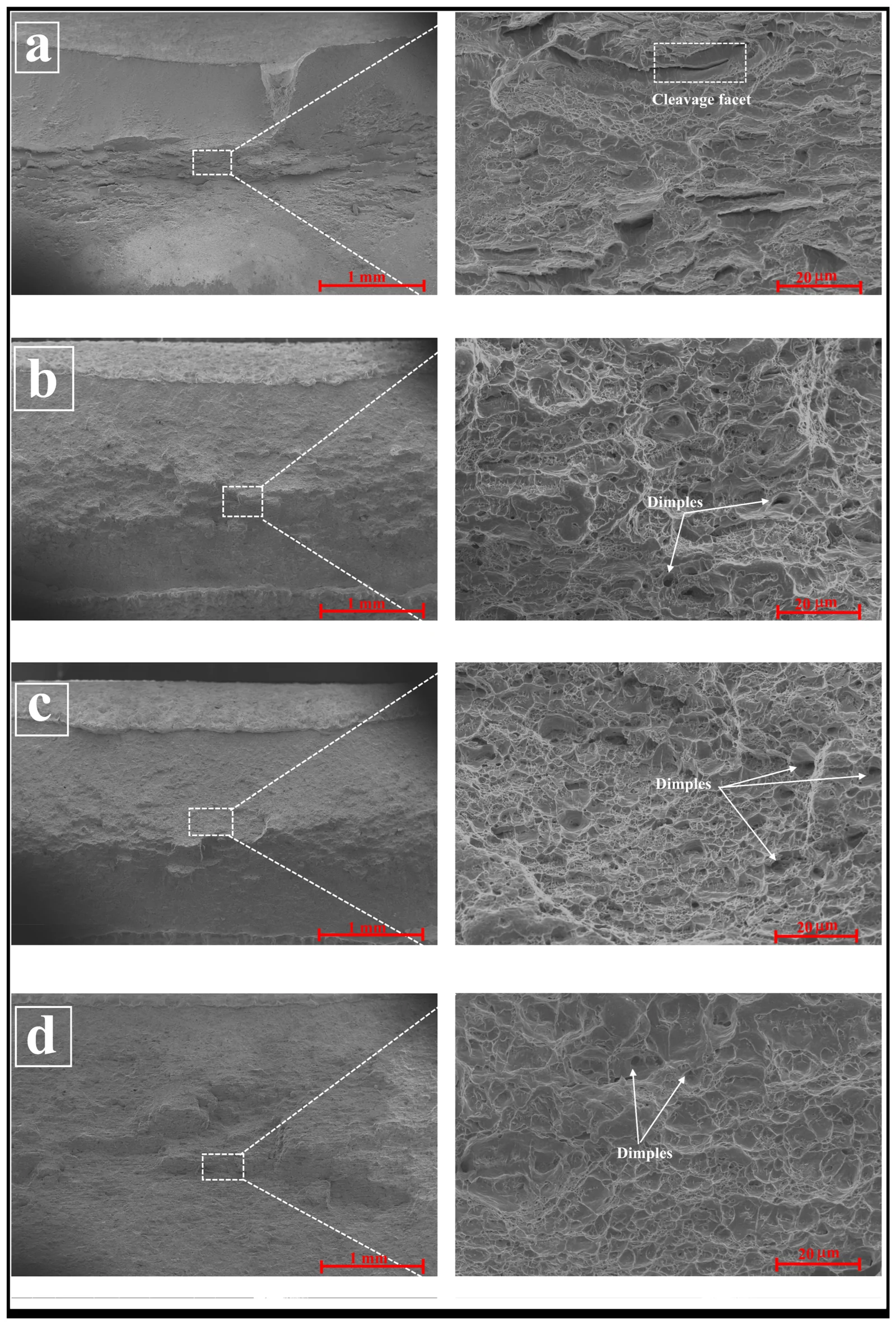
SEM images of the fracture surfaces of Hardox 500 Tuf specimens: (**a**) 23 °C, (**b**) AC 800 °C, (**c**) BC 800 °C and (**d**) WSC 800 °C. Left panels: 100×, 1-mm scale bars; right panels: 4000×, 20-µm scale bars.

Figure 19 and Figure 20 show the fracture surfaces of Hardox 450 and Hardox 500 Tuf, respectively, at 23 °C and after exposure to 800 °C under each cooling procedure. The low- and high-magnification panels in Figure 19 and Figure 20 show the fracture-surface overview and a selected local region, respectively. The as-received specimens show a mixed morphology, in which dimpled regions are accompanied by flat, facet-like areas marked as cleavage facets; a similar combination of ductile dimples with few cleavage facets has been reported for Hardox 450 [18]. After 800 °C, the examined regions are covered mainly by dimples, the characteristic feature of ductile fracture by microvoid nucleation, growth and coalescence [16,24], and the BC specimens show locally coarser dimples than the AC and WSC specimens. This change is consistent with the tensile response: under AC and BC, the strain at maximum stress is roughly six to ten times the reference value.

The fractography thus complements the tensile measurements by showing how the examined regions failed, while the tensile curves describe how deformation accumulated before failure. The images do not provide direct evidence of grain growth or quantify the phases responsible for softening. Moreover, observations at 800 °C cannot establish the fracture mechanism of the 1000 °C WSC specimens. These distinctions allow the mechanical and microscopy results to be considered together without assigning more diagnostic power to the fracture surfaces than they provide.

Taken together, the tests establish a distinction between residual tensile response and local post-fracture observations. They do not resolve which phase mixture produces the high WSC strength at 1000 °C. That question requires characterization at the same temperature and cooling conditions, preferably including undeformed companion specimens and quantitative phase or metallographic measurements.

## 4. Empirical Representation of Residual Properties

A continuous residual-factor formulation summarizes the measured post-fire tensile properties and local hardness of Hardox 450 and Hardox 500 Tuf. It is an empirical description of this dataset, not an independently validated predictive relationship or a design rule.

For a given property (X), the normalized residual factor is defined as:

(1) $$R_{X}\left(T\right)=\frac{X_{T}}{X_{23}}$$

where *R_X_*(*T*) is the normalized residual property after exposure to peak temperature *T* and subsequent cooling; *X_T_* is the property measured at room temperature after that treatment; and *X*_23_ is the corresponding value for the unheated 23 °C reference specimen. Thus, *T* denotes the prior exposure temperature, not the temperature of the mechanical test. In this study, X represents E, *f_y_*, *f_u_*, *ε_u_*, or HV.

A continuous piecewise linear representation with breakpoints at 300, 600 and 800 °C is used to summarize the changes in the measured trends (Equation (2)):

(2) $$R_{X}\left(T\right)=\left\{\begin{array}{ll} 1+a_{X}\left(T-23\right), & \;\mathrm{for}\;23\leq T\leq 300\\ 1+a_{X}\left(T-23\right)+b_{X}\left(T-300\right), & \;\mathrm{for}\;300<T\leq 600\\ 1+a_{X}\left(T-23\right)+b_{X}\left(T-300\right)+c_{X}\left(T-600\right), & \;\mathrm{for}\;600<T\leq 800\\ 1+a_{X}\left(T-23\right)+b_{X}\left(T-300\right)+c_{X}\left(T-600\right)+d_{X}\left(T-800\right), & \;\mathrm{for}\;800<T\leq 1000 \end{array}\right.$$

where *a_X_*, *b_X_*, *c_X_* and *d_X_* are the calibrated coefficients for the selected property, steel grade, and cooling condition. Their values are given in Table 2. Equation (2) is continuous at 300, 600 and 800 °C: the slopes in its four intervals are *a_X_*, *a_X_* + *b_X_*, *a_X_* + *b_X_* + *c_X_* and *a_X_* + *b_X_* + *c_X_* + *d_X_*. Accordingly, *b_X_*, *c_X_* and *d_X_* represent changes in slope at the transition temperatures, rather than the independent slopes of the later intervals.

The breakpoints at 300, 600 and 800 °C are empirical choices, not measured phase-transformation temperatures. The first breakpoint is placed at 300 °C because the measured strength factors remain close to unity up to that temperature and fall steeply above it; a single segment between 23 and 400 °C underestimates the 300 °C strength factors by approximately 0.10. For the modulus, proof-stress, UTS and hardness factors, the coefficients are obtained by least squares subject to the constraint *R_X_*(*T*) ≤ 1 over 23–1000 °C, so that the fitted curve never exceeds the unheated reference. Measured factors above unity occur only for the modulus, where individual values reach 1.20; they are treated as scatter and are not followed by the model. The strain factor is not constrained, because the strain at maximum stress increases after exposure. Equation (2) satisfies *R_X_*(23) = 1. To obtain a property, the row for the relevant steel, cooling method and residual factor is selected from Table 2, Equation (2) is evaluated at the prior exposure temperature, and the resulting factor is multiplied by *X*_23_ using Equation (1). For example, the Hardox 500 Tuf–WSC UTS coefficients give *R_fu_*(1000) = 1 − 0.000217(977) − 0.001312(700) + 0.001301(400) + 0.001996(200) ≈ 0.789; with *f_u,_*_23_ ≈ 1579 MPa, this corresponds to approximately 1246 MPa. Minor differences arise from rounding the tabulated coefficients.

The curves in Figure 6, Figure 7, Figure 8, Figure 9, Figure 10, Figure 11, Figure 12, Figure 13, Figure 16 and Figure 17 describe the calibration observations and should not be interpreted as validation. Each steel–cooling–property series contains an anchored reference and six heated observations, with four coefficients. The 800 and 1000 °C observations each lie at the end of a segment with their own coefficients, so *c_X_* and *d_X_* reproduce these two points exactly unless the upper bound of unity is active, without independent information on the intervening response; the remaining two coefficients are fitted to the four observations between 300 and 600 °C. Agreement at 800 and 1000 °C therefore does not demonstrate predictive ability. Table 2 reports the root-mean-square error (RMSE) and coefficient of determination (R^2^) of each fit over the six heated observations. The RMSE is 0.01–0.05 for the proof-stress, UTS and hardness factors, with a largest single deviation of 0.08; it is 0.03–0.08 for the modulus factors, with a largest deviation of 0.20, and 0.13–0.46 for the strain factors, whose values range up to approximately 11. Leave-one-out cross-validation is possible only for the four observations between 300 and 600 °C; it gives prediction errors of 0.01–0.10 for the proof-stress, UTS and hardness factors, 0.04–0.13 for the modulus factors and 0.40–1.26 for the strain factors, which are up to several times the fitting errors. The equations are therefore descriptive summaries, and the strain-factor curves in particular should not be used for interpolation. The modulus-factor fits summarize values that lie within the measurement scatter described in Section 3.2. No independent validation is claimed.

The equations apply only as descriptive summaries for the investigated 4-mm coupons, nominal 20 °C/min heating rate, 30-min hold and stated cooling procedures. They should not be extrapolated outside 23–1000 °C or transferred directly to components with different thicknesses, temperature gradients, loading or restraint. Unknown specimen cooling histories, post-deformation hardness sampling and the absence of 1000 °C microstructural characterization further limit interpretation. Replicated tests with measured cooling histories and independent validation are required before engineering prediction or reuse decisions.

## 5. Conclusions

The investigation identifies the following specimen-level trends for 4-mm Hardox 450 and Hardox 500 Tuf under the thermal programme examined:

➢ Both grades retain most of their reference UTS after 300 °C exposure, whereas retention falls to approximately 50–51% at 600 °C. Differences between cooling conditions at 600 °C are small in the observed data, but statistical equivalence is not established.
➢ After 1000 °C exposure, WSC gives higher residual UTS than AC or BC, with retention of approximately 52.5% for Hardox 450 and 79% for Hardox 500 Tuf. The corresponding strains at maximum stress are only 3.50% and 3.79%, showing that the higher strength is accompanied by reduced strain capacity relative to the corresponding AC and BC specimens.
➢ Local hardness after 1000 °C WSC is approximately 48% of the reference value in both grades. Its limited recovery, despite the higher tensile strength, shows that these properties must be assessed separately. Near-fracture, post-tensile microhardness does not establish bulk hardness, abrasion resistance or remaining service life.
➢ XRD and SEM characterize the reference and 800 °C conditions qualitatively and do not establish the phase mechanism at 1000 °C. The residual-factor equations summarize the measured trends, but do not establish predictive performance beyond the investigated thermal programme.

## Figures and Tables

**Table 1:** Chemical compositions reported in the manufacturer’s inspection certificates (wt.%). The values are not means of repeated chemical analyses in this study; analytical uncertainty is not reported here.

Steel Grade	C	Si	Mn	P	S	Cr	Ni	Mo	V	Ti	Cu	Al	Nb	B	N	CEV
Hardox 450	0.177	0.18	1.25	0.012	0.001	0.24	0.10	0.04	0.01	0.01	0.01	0.037	0.001	0.0012	0.003	0.45
Hardox 500 Tuf	0.249	0.19	0.20	0.006	0.001	0.38	0.49	0.05	0.01	0.003	0.13	0.054	0.001	0.0012	0.002	0.41

**Table 2:** Coefficients of the empirical residual-factor equations for Hardox 450 and Hardox 500 Tuf.

Hardox Type	Cooling	Residual Factor	*a_X_* (°C^−1^)	*b_X_* (°C^−1^)	*c_X_* (°C^−1^)	*d_X_* (°C^−1^)	RMSE	R^2^
Hardox 450	AC	*E_T_*/*E*_23_	0.000000	0.000000	−0.000119	−0.000742	0.033	0.849
Hardox 450	AC	*f_y_*_,_*_T_*/*f_y_*_,23_	−0.000154	−0.001470	0.000871	0.000299	0.030	0.986
Hardox 450	AC	*f_u_*_,_*_T_*/*f_u_*_,23_	−0.000249	−0.001266	0.001054	0.000103	0.031	0.981
Hardox 450	AC	*ε_u_*_,_*_T_*/*ε_u_*_,23_	−0.001135	0.010544	0.023797	−0.035496	0.463	0.985
Hardox 450	AC	*H_v_*_,_*_T_*/*H_v_*_,23_	−0.000457	−0.000585	0.000710	0.000257	0.020	0.980
Hardox 450	WSC	*E_T_*/*E*_23_	0.000000	0.000000	−0.000243	−0.000686	0.084	0.574
Hardox 450	WSC	*f_y_*_,_*_T_*/*f_y_*_,23_	0.000000	−0.001726	0.000554	0.001859	0.045	0.972
Hardox 450	WSC	*f_u_*_,_*_T_*/*f_u_*_,23_	−0.000021	−0.001687	0.001738	0.000158	0.041	0.956
Hardox 450	WSC	*ε_u_*_,_*_T_*/*ε_u_*_,23_	0.000463	0.007468	0.029457	−0.082608	0.401	0.986
Hardox 450	WSC	*H_v_*_,_*_T_*/*H_v_*_,23_	−0.000474	−0.000452	0.000776	−0.000272	0.013	0.991
Hardox 450	BC	*E_T_*/*E*_23_	0.000000	0.000000	0.000000	−0.001060	0.076	0.564
Hardox 450	BC	*f_y_*_,_*_T_*/*f_y_*_,23_	−0.000018	−0.001684	0.000836	0.000395	0.019	0.995
Hardox 450	BC	*f_u_*_,_*_T_*/*f_u_*_,23_	−0.000094	−0.001522	0.001039	0.000173	0.020	0.993
Hardox 450	BC	*ε_u_*_,_*_T_*/*ε_u_*_,23_	0.000517	0.007932	0.023005	−0.027572	0.256	0.996
Hardox 450	BC	*H_v_*_,_*_T_*/*H_v_*_,23_	−0.000244	−0.000984	0.001102	−0.000139	0.005	0.999
Hardox 500 Tuf	AC	*E_T_*/*E*_23_	−0.000264	0.000507	−0.001239	0.001041	0.033	0.885
Hardox 500 Tuf	AC	*f_y_*_,_*_T_*/*f_y_*_,23_	−0.000049	−0.001614	0.000462	0.001132	0.027	0.991
Hardox 500 Tuf	AC	*f_u_*_,_*_T_*/*f_u_*_,23_	−0.000198	−0.001386	0.000822	0.000867	0.028	0.986
Hardox 500 Tuf	AC	*ε_u_*_,_*_T_*/*ε_u_*_,23_	−0.000656	0.003107	0.020280	−0.032993	0.129	0.996
Hardox 500 Tuf	AC	*H_v_*_,_*_T_*/*H_v_*_,23_	−0.000599	−0.000491	0.000911	−0.000030	0.017	0.987
Hardox 500 Tuf	WSC	*E_T_*/*E*_23_	−0.000218	0.000345	−0.000437	−0.000003	0.036	0.568
Hardox 500 Tuf	WSC	*f_y_*_,_*_T_*/*f_y_*_,23_	−0.000062	−0.001534	0.000228	0.003087	0.021	0.992
Hardox 500 Tuf	WSC	*f_u_*_,_*_T_*/*f_u_*_,23_	−0.000217	−0.001312	0.001301	0.001996	0.022	0.986
Hardox 500 Tuf	WSC	*ε_u_*_,_*_T_*/*ε_u_*_,23_	−0.000901	0.003772	0.001712	−0.011592	0.142	0.941
Hardox 500 Tuf	WSC	*H_v_*_,_*_T_*/*H_v_*_,23_	−0.000382	−0.000901	0.001159	0.000100	0.038	0.943
Hardox 500 Tuf	BC	*E_T_*/*E*_23_	−0.000154	−0.000089	0.000255	0.000376	0.044	0.335
Hardox 500 Tuf	BC	*f_y_*_,_*_T_*/*f_y_*_,23_	−0.000034	−0.001562	0.000336	0.001059	0.005	1.000
Hardox 500 Tuf	BC	*f_u_*_,_*_T_*/*f_u_*_,23_	−0.000186	−0.001348	0.000734	0.000792	0.011	0.998
Hardox 500 Tuf	BC	*ε_u_*_,_*_T_*/*ε_u_*_,23_	−0.000777	0.003452	0.023247	−0.036493	0.132	0.996
Hardox 500 Tuf	BC	*H_v_*_,_*_T_*/*H_v_*_,23_	−0.000668	−0.000362	0.000731	0.000211	0.011	0.994

Note: AC = air cooling, WSC = water spray cooling, and BC = blanket cooling. The residual factors are normalized with respect to the corresponding 23 °C reference values. RMSE and R^2^ refer to the six heated observations of each series.

## Data Availability

The raw data supporting the conclusions of this article will be made available by the authors on request.
